# RTTAP: Empowering metatranscriptomic data analysis with a read‐based total‐infectome taxonomic solution

**DOI:** 10.1002/imo2.70044

**Published:** 2025-07-29

**Authors:** Wei Jiang, Herui Liao, Mang Shi, Liangjun Chen, Yanni Sun

**Affiliations:** ^1^ Department of Electrical Engineering City University of Hong Kong, Tat Chee Avenue Kowloon Hong Kong China; ^2^ State Key Laboratory for Biocontrol, Shenzhen Key Laboratory of Systems Medicine for Inflammatory Diseases, School of Medicine Shenzhen campus of Sun Yat‐sen University, Sun Yat‐sen University Shenzhen China; ^3^ Department of Laboratory Medicine Zhongnan Hospital of Wuhan University Wuhan China

## Abstract

Metatranscriptomic data analysis is a complex task due to its sheer volume and the need for sophisticated bioinformatics tools. To address this, we developed the Read‐based Total‐infectome Taxonomic Analysis Pipeline (RTTAP), an automated pipeline for metatranscriptomic data analysis that eliminates the need for users to manually select databases, tools, or parameters. RTTAP provides a comprehensive solution for “total‐infectome” analysis, enabling simultaneous detection of viruses, bacteria, and fungi. Additionally, RTTAP delivers detailed functional profiling of antibiotic resistance genes (ARGs) and high‐resolution viral strain analysis, offering researchers a powerful tool for advanced metatranscriptomic studies. The pipeline's performance was validated using both simulated and real clinical metatranscriptomic datasets, demonstrating high accuracy in taxonomic classification and relative abundance estimation.

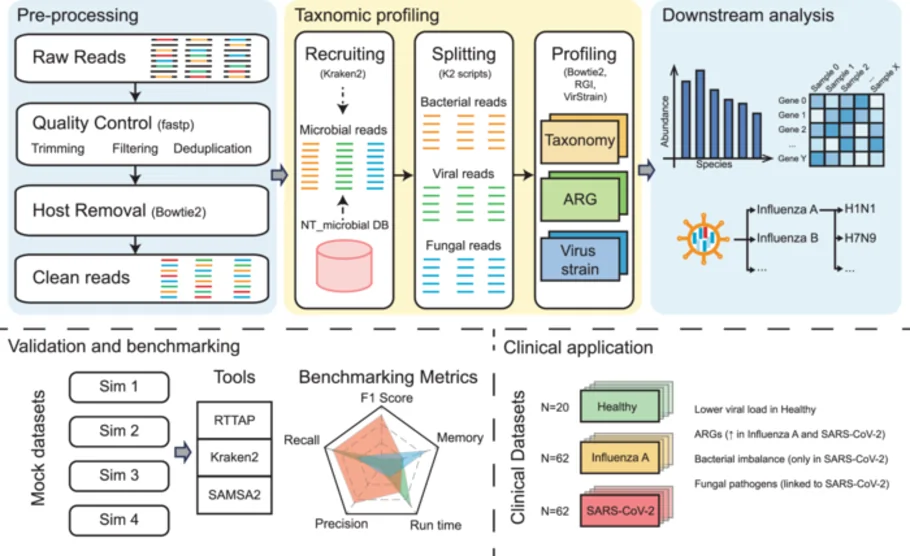

## AUTHOR CONTRIBUTIONS

**Wei Jiang**: Conceptualization; data curation; methodology; software; investigation; validation; formal analysis; visualization; Writing—original draft; Writing—review and editing. **Herui Liao**: Conceptualization; data curation; methodology; formal analysis; software. **Mang Shi**: Formal analysis; investigation; project administration; resources. **Liangjun Chen**: Conceptualization; methodology; data curation; investigation; validation; formal analysis; funding acquisition; project administration; resources; Writing—review and editing. **Yanni Sun**: Conceptualization; formal analysis; funding acquisition; project administration; resources; Writing—review and editing; validation; supervision.

## CONFLICT OF INTEREST STATEMENT

The authors declare no conflicts of interest.

## ETHICS STATEMENT

This study was approved by the Ethics Committee of the Zhongnan Hospital, Wuhan University (No. 2021024) and the Ethics Committee of Sun Yat‐sen University (No. 2020034).

To the Editor,

Metatranscriptomic and metagenomic analyses have become essential tools for studying microbial communities in a wide range of environments, particularly within the human microbiome [1, 2, 3]. Despite their importance, these approaches face three key computational challenges: (1) inherent limitations of composition analysis in current classification algorithms, (2) trade‐offs in selecting appropriate reference databases, and (3) inefficiencies in the integration of existing analytical pipelines. Together, these challenges impede the accuracy and efficiency of microbial profiling, posing significant obstacles in clinical settings where timely and reliable results are critical.

Existing alignment and mapping‐based tools, such as BLAST [4] and Bowtie2 [5], are computationally expensive and time‐consuming due to their exhaustive search algorithms. Kmer‐based methods, such as Kraken2 [6], achieve faster processing but incur false positives from short k‐mer matches (typically 31–35 bp). Furthermore, marker gene‐based tools such as MetaPhlAn4 [7] often lack specificity and fail to detect viruses.

Database selection introduces additional constraints. While large databases like NCBI NR/NT [8] require substantial storage and search times, specialized databases such as GTDB [9] frequently lack comprehensive viral coverage, limiting their utility for total‐infectome profiling.

Current pipelines exemplify these trade‐offs: MetaPro [10] employs a comprehensive assembly‐based approach, requiring up to 17 distinct reference databases totaling over 700GB in storage. While this enables detailed taxonomic classification, it creates substantial computational burdens. In contrast, SAMSA2 [11] implements a lightweight design that relies solely on DIAMOND [12] alignments for taxonomic classification, significantly reducing resource requirements but remaining subject to DIAMOND's inherent database limitations and alignment constraints.

To address these multidimensional challenges, we developed RTTAP (Read‐based Total‐infectome Taxonomic Analysis Pipeline), a user‐friendly yet comprehensive solution for the microbial taxonomic analysis of metatranscriptomic samples. RTTAP integrates stringent preprocessing steps and parallel detection of bacteria, viruses, and fungi. Unlike standard tools, RTTAP incorporates key optimizations to improve accuracy and clinical relevance, such as customizable abundance filters and taxonomic refinement via a lowest common ancestor (LCA) algorithm. Additionally, RTTAP extends beyond taxonomy by enabling the detection of antimicrobial resistance genes (ARGs) and strain‐level viral characterization, supporting actionable insights in clinical settings.

We benchmarked RTTAP against Kraken2, a widely‐used taxonomic classifier, and SAMSA2, a metatranscriptomic analysis pipeline, using simulated datasets. Performance was assessed using the F1 score—a metric that balances precision and recall, ranging from 0 to 1, with higher values indicating greater classification accuracy. RTTAP outperformed both tools, achieving F1‐scores of 0.87 at the genus level and 0.84 at the species level, demonstrating improved sensitivity and specificity. When applied to clinical samples, RTTAP maintained robust performance in detecting real‐world pathogens, underscoring its practical utility in both diagnostic and research contexts.

### Overview of RTTAP

**Figure 1:**
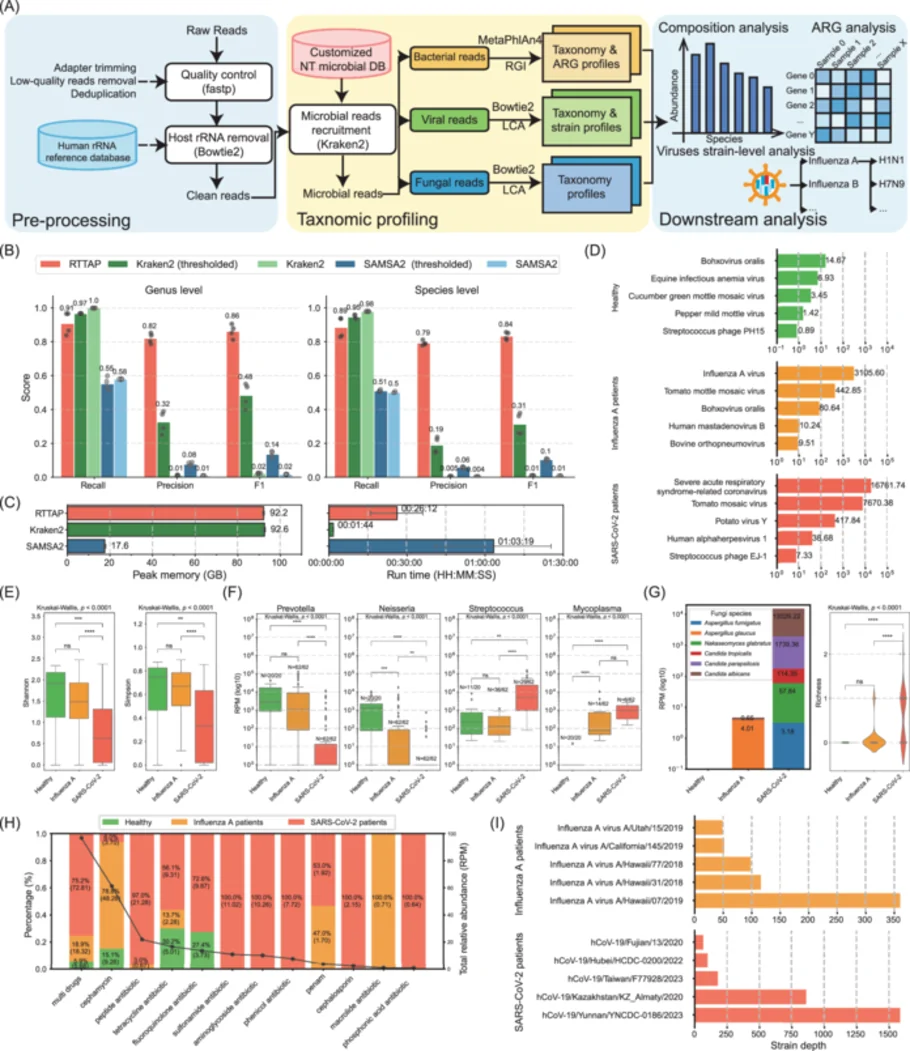
Workflow and performance of RTTAP. (A) Sketch of RTTAP, including three stages illustrated in colored blocks, with each name labeled at the bottom left. LCA: lowest‐common ancestor algorithm. ARG: antibiotic resistance genes. (B) Performance comparison of RTTAP, Kraken2, and SAMSA2. Bars represent mean values for each metric (numerically labeled); dots indicate results from four simulated datasets. (C) Computational resource usage (peak memory and run time) for the benchmarked tools. Bars denote mean values. (D) Top 5 viral species across sample groups. *X*‐axis: log~10~(reads per million, RPM); *Y*‐axis: viral species. Groups are color‐coded and labeled on the left. (E) Bacterial alpha diversity. Left: Shannon index; Right: Simpson index. (F) Relative abundance of bacterial species disrupted after Influenza A or SARS‐CoV‐2 infection. Species names are labeled above each plot. *N* values represent the number of sample sizes. (G) Fungal community analysis. Left: Taxonomic composition; Right: richness (alpha diversity). (H) Distribution of ARG drug‐resistant types. *X*‐axis: ARG drug‐resistant types; *Y*‐axis: Relative abundance (%, with RPM values labeled). Line plot shows total RPM of groups per ARG type. (I) Top 5 viral strains detected in Influenza A and SARS‐CoV‐2 samples. Group names are attached on the left. Statistical tests: For panels (E–G), significance was assessed using the Kruskal–Wallis test and Dunn's post hoc test. Significance levels: ns (*p* > 0.05), *(*p* ≤ 0.05), **(*p* ≤ 0.01), ***(*p* ≤ 0.001), ****(*p* ≤ 0.0001).

Our method, RTTAP, streamlines analysis through a three‐stage structure, as illustrated in Figure 1A. Initially, it performs basic quality control steps and removes host‐originated sequences by mapping reads to human ribosomal RNA reference sequences. Subsequently, it employs a rapid, read‐based taxonomic classifier to categorize the reads into three distinct groups: viruses, bacteria, and fungi. For each group, it leverages specialized tools and curated databases to conduct a more detailed and precise analysis. The final stage integrates functions for ARGs detection and viral strain profiling. More details of each stage are shown in Supporting Information: Section A.

### Validation and performance of RTTAP

In real‐world applications, taxonomic classifiers are typically paired with their recommended reference databases. Since the choice of database plays a critical role in determining the performance of these tools—and prior research [13] has identified Kraken2 and Kaiju as optimal for nucleotide‐ and protein‐based classification methods, respectively—we conducted a benchmarking analysis with Kraken2 and Kaiju. This analysis evaluated multiple tool‐database combinations, using Kraken2 and Kaiju with both their standard reference databases and customized databases. This comparative analysis aimed to identify the optimal pipeline configuration for the initial microbial read recruitment stage.

Experiment details are explained in the Supporting Information: Section B. Based on our experimental results, Kraken2 paired with the customized nt_microbial database outperforms other combinations with its high recall (Figure S1), prediction rate (Figure S2), and fast processing speed (Figure S3), being the optimal choice for the initial microbial read recruitment stage of RTTAP. Notably, all the combinations demonstrated poor performance in category precision (smaller than 0.05), indicating a high rate of false‐positive classifications (Figure S1). Consequently, the F1 scores were also small. These results suggested that the tested taxonomic classifiers require complementary false‐positive reduction strategies, as their outputs contain an excessive number of false‐positive results, severely compromising classification reliability. However, through careful optimization of downstream processes, RTTAP achieves high precision across all experiments, highlighting its effectiveness and reliability in accurate taxonomic classification.

Having established Kraken2 with the nt_microbial database as the optimal initial classifier, we next evaluated the comprehensive performance of the full RTTAP pipeline. This evaluation was conducted on four simulated datasets, assessing classification accuracy across four distinct categories: viruses, bacteria, fungi, and overall performance (Figure S4).

We further conducted comprehensive benchmarking of RTTAP against two established approaches: Kraken2 (a taxonomic classifier) and SAMSA2 (a metatranscriptomic pipeline). All evaluations were performed using the same simulated datasets to ensure consistency. Although RTTAP incorporates Kraken2 for initial taxonomic classification, our results demonstrate that the complete pipeline outperforms standalone Kraken2 (Figure 1B). This improvement is largely attributed to RTTAP's downstream validation steps, which effectively reduce false‐positive predictions generated during the initial classification.

The notably lower precision and F1 scores observed for both Kraken2 and SAMSA2 are primarily attributable to a high rate of false‐positive predictions, particularly at lower taxonomic levels such as genus and species. SAMSA2 also exhibits substantially lower recall, likely due to its primary focus on bacterial taxa while neglecting viruses and fungi. Despite this, its low precision—similar to that of Kraken—suggests a comparable tendency to generate false positives. In contrast, RTTAP integrates Bowtie2, which is known for its speed and alignment accuracy, making it well‐suited for fine‐grained, lower‐level classification. Additionally, the use of an LCA‐based taxonomic assignment strategy further enhances accuracy by minimizing erroneous classifications. Together, these optimizations significantly improve classification precision and contribute to an overall increase in F1 score.

To ensure a fair comparison, we uniformly applied RTTAP's abundance threshold filtering to all tools' outputs. Benchmarking results confirmed that applying an abundance threshold consistently improved classification performance across tools. Specifically, thresholding led to F1 score improvement of 0.47 for Kraken2 and 0.12 for SAMSA2 at the genus level, primarily by reducing false positives while maintaining comparable recall (Figure 1B). These findings underscore the importance of abundance filtering in optimizing the precision‐recall trade‐off in metatranscriptomic classification. Importantly, RTTAP allows users to adjust abundance thresholds based on their specific data characteristics and analytical goals.

Regarding the error evaluation of our pipeline, we would like to clarify that “1‐precision” corresponds to the Type I error, representing the proportion of false‐positive categories—that is, categories predicted by the pipeline that do not actually exist in the sample. Similarly, “1‐recall” reflects the Type II error, indicating the proportion of false negatives, where existing categories are missed during classification.

Additionally, we present computational performance metrics for all benchmarked tools, including peak memory usage and runtime analysis (Figure 1C). Since RTTAP incorporates Kraken2 for classification, its peak memory usage is nearly identical to that of Kraken2. However, the runtime is longer due to additional downstream processing. Nevertheless, RTTAP still demonstrates competitive speed for taxonomic analysis.

### Application to clinical samples

In addition, RTTAP was applied to analyze a total of 144 clinical samples to demonstrate its functionalities and performance. Taxonomic profiles of all samples were generated and visualized using Python scripts. We provide preliminary interpretations of these clinical results below, with additional analyses available in the Supporting Information: Section C.

**Taxonomic profiles.** We present the top‐5 abundant viruses in these samples in Figure 1D. Influenza A virus and severe acute respiratory syndrome‐related coronavirus are the dominant viruses in Influenza A patients and SARS‐CoV‐2 patients, respectively. At the same time, healthy individuals show a much lower virus abundance than these respiratory disease patients. The detection of plant viruses in these samples should be considered normal, given frequent dietary exposure through fruit and vegetable consumption, as previously reported [14].

Regarding bacterial communities, SARS‐CoV‐2 patients show significantly lower bacteriome diversity (Kruskal–Wallis test, *p* < 0.0001; Dunn's post hoc test, *p* < 0.01) compared to healthy and Influenza A patients (Figure 1E), indicating their potential disruption in respiratory bacteriome. Specifically, *Prevotella* abundance was markedly reduced in SARS‐CoV‐2 patients, while *Neisseria* showed significant depletion in both viral infection groups (Kruskal–Wallis test, *p* < 0.0001; Dunn's post hoc test, *p* < 0.01; Figure 1F *Prevotella*, *Neisseria*). Conversely, *Streptococcus* demonstrated increased abundance in SARS‐CoV‐2‐positive cases (Kruskal–Wallis test, *p* < 0.0001; Dunn's post hoc test, *p* < 0.01; Figure 1F *Streptococcus*). Notably, we observed elevated levels of opportunistic pathogens like *Mycoplasma* in both Influenza A and SARS‐CoV‐2 groups (Kruskal–Wallis test, *p* < 0.0001; Dunn's post hoc test, *p* < 0.0001; Figure 1F *Mycoplasma*), suggesting possible viral‐bacterial co‐infections.

In contrast, SARS‐CoV‐2 patients tend to have more abundant and diverse fungi communities compared to the other two groups (Figure 1G), which suggests SARS‐CoV‐2 patients are more likely to have coinfection with fungi, especially *Candida albicans*, and require special attention in clinical cases. We presented a full view of the taxonomic profiles at both the genus and species levels in Figures S5 and S6, respectively, and the detailed data can be found in Table S1. The comprehensive total‐infectome taxonomic analysis employed in this study offers a broader perspective than single‐omics approaches, providing deeper insights into the intricate relationships and potential correlations among various biological factors.

**ARG profiles.** The abundance comparison of different drug‐resisting ARGs is depicted in Figure 1H. SARS‐CoV‐2 patients exhibited the highest abundance of ARGs across nearly all categories, and contained most of the multidrug‐resistant genes. Influenza A patients presented the most *Cephamycin* and *Macrolide antibiotic*‐resisting ARGs. Interestingly, healthy individuals were found to carry detectable ARGs, particularly those conferring resistance to *cephamycins*, *tetracyclines*, *fluoroquinolones*, as well as multidrug‐resistant variants.

The complete ARG composition across all clinical samples is shown in Figure S7, with detailed data available in Table S2. Notably, we observed multiple instances of ARGs co‐occurring with their known bacterial hosts. Sample W48‐04‐Y‐d36 from the SARS‐CoV‐2 cohort exhibited the highest number of co‐occurrences (13 ARG‐bacterium pairs), suggesting a potentially elevated risk of antibiotic resistance dissemination during the sampling period. At the individual ARG level, *CfxA2* demonstrated the highest frequency of co‐occurrence (8 instances), suggesting its potentially widespread role in antibiotic resistance dissemination. These RTTAP‐generated ARG profiles not only characterize resistance determinants within the samples but may also inform clinical decision‐making regarding antibiotic therapy selection.

**Viruses strain‐level profiles.** By utilizing the viral strain‐level analysis incorporated in RTTAP, we presented the top‐5 abundant viral strains in Figure 1I. The viral strain‐level profiles of the 144 clinical samples are illustrated in Figure S8 and the detailed data can be found in Table S3. These strain‐level profiles of viruses illustrated the presence and abundance of different virus strains, thus providing useful information regarding virus source tracking and high‐virulent viral strain detection and treatment.

RTTAP represents an efficient and sensitive taxonomic analysis pipeline specifically optimized for metatranscriptomic infectome profiling, encompassing viruses, bacteria, and fungi. By leveraging a reference‐based classification strategy with Kraken2 and the nt_microbial database, the pipeline achieves high accuracy in microbial detection while overcoming limitations associated with assembly‐based methods, such as reduced sensitivity for low‐abundance taxa and excessive computational demands. Validation using simulated and clinical datasets confirmed its ability to reliably characterize microbial communities, antibiotic resistance genes, and viral strains, demonstrating its utility in both research and clinical diagnostics.

The pipeline's user‐friendly design ensures accessibility for researchers with varying levels of bioinformatics expertise, balancing simplicity for routine analyses with customizable options for advanced applications. However, the current exclusion of protists represents a limitation in certain infection contexts, warranting future database expansions to improve taxonomic coverage. Further enhancements, including algorithmic optimizations and machine learning integration, may further refine detection sensitivity and computational efficiency.

Overall, RTTAP provides a robust and scalable framework for metatranscriptomic infectome analysis, with immediate applications in pathogen surveillance and clinical decision‐making, as well as strong potential for future refinements to broaden its scope and performance.

Detailed procedures for the workflow of RTTAP and data preparation are available in the Supporting Information.

## Supporting information

Supporting information.

Supporting information.

## Data Availability

The pipeline RTTAP, Python scripts for visualization, and necessary databases are available at https://github.com/weijiang34/RTTAP. The raw sequence data reported in this paper have been deposited in the National Genomics Data Center (GSA: CRA016815) that are publicly accessible at https://ngdc.cncb.ac.cn/gsa/browse/CRA016815. Supplementary materials (methods, figures, tables, graphical abstract, data sources, slides, videos, Chinese translated version, and update materials) may be found in the online DOI or iMetaOmics http://www.imeta.science/imetaomics/.

## References

[1] Miao, Qing , Tianzhu Liang , Na Pei , Chunjiao Liu , Jue Pan , Na Li , Qingqing Wang , et al. 2022. “Evaluation of Respiratory Samples in Etiology Diagnosis and Microbiome Characterization by Metagenomic Sequencing.” Respiratory Research 23: 345. 10.1186/s12931-022-02230-3 PMC9748891 36517824

[2] Ojala, Teija , Esko Kankuri , and Matti Kankainen . 2023. “Understanding Human Health Through Metatranscriptomics.” Trends in Molecular Medicine 29: 376–389. 10.1016/j.molmed.2023.02.002 36842848

[3] Rajagopala, Seesandra V. , Nicole G. Bakhoum , Suman B. Pakala , Meghan H. Shilts , Christian Rosas‐Salazar , Annie Mai , Helen H. Boone , et al. 2021. “Metatranscriptomics to Characterize Respiratory Virome, Microbiome, and Host Response Directly From Clinical Samples.” Cell Reports Methods 1: 100091. 10.1016/j.crmeth.2021.100091 PMC8594859 34790908

[4] Altschul, Stephen F. , Warren Gish , Webb Miller , Eugene W. Myers , and David J. Lipman . 1990. “Basic Local Alignment Search Tool.” Journal of Molecular Biology 215: 403–410. 10.1016/S0022-2836(05)80360-2 2231712

[5] Langmead, Ben , and Steven L. Salzberg . 2012. “Fast Gapped‐Read Alignment With Bowtie 2.” Nature Methods 9: 357–359. 10.1038/nmeth.1923 PMC3322381 22388286

[6] Wood, Derrick E. , Jennifer Lu , Ben Langmead . 2019. “Improved Metagenomic Analysis With Kraken 2.” Genome Biology 20: 257. 10.1186/s13059-019-1891-0 PMC6883579 31779668

[7] Blanco‐Míguez, Aitor , Francesco Beghini , Fabio Cumbo , Lauren J. McIver , Kelsey N. Thompson , Moreno Zolfo , Paolo Manghi , et al. 2023. “Extending and Improving Metagenomic Taxonomic Profiling With Uncharacterized Species Using MetaPhlAn 4.” Nature Biotechnology 41: 1633–1644. 10.1038/s41587-023-01688-w PMC10635831 36823356

[8] NCBI Resource Coordinators . 2014. “Database Resources of the National Center for Biotechnology Information.” Nucleic Acids Research 42: D7–D17. 10.1093/nar/gkt1146 PMC3965057 24259429

[9] Parks, Donovan H. , Maria Chuvochina , Christian Rinke , Aaron J. Mussig , Pierre‐Alain Chaumeil , and Philip Hugenholtz . 2022. “GTDB: An Ongoing Census of Bacterial and Archaeal Diversity Through a Phylogenetically Consistent, Rank Normalized and Complete Genome‐Based Taxonomy.” Nucleic Acids Research 50: D785–D794. 10.1093/nar/gkab776 PMC8728215 34520557

[10] Taj, Billy , Mobolaji Adeolu , Xuejian Xiong , Jordan Ang , Nirvana Nursimulu , and John Parkinson . 2023. “MetaPro: A Scalable and Reproducible Data Processing and Analysis Pipeline for Metatranscriptomic Investigation of Microbial Communities.” Microbiome 11: 143. 10.1186/s40168-023-01562-6 PMC10294448 37370188

[11] Westreich, Samuel T. , Michelle L. Treiber , David A. Mills , Ian Korf , and Danielle G. Lemay . 2018. “SAMSA2: A Standalone Metatranscriptome Analysis Pipeline.” BMC Bioinformatics 19: 175. 10.1186/s12859-018-2189-z PMC5963165 29783945

[12] Buchfink, Benjamin , Klaus Reuter , and Hajk‐Georg Drost . 2021. “Sensitive Protein Alignments at Tree‐of‐Life Scale Using DIAMOND.” Nature Methods 18: 366–368. 10.1038/s41592-021-01101-x PMC8026399 33828273

[13] Ye, Simon H. , Katherine J. Siddle , Daniel J. Park , and Pardis C. Sabeti . 2019. “Benchmarking Metagenomics Tools for Taxonomic Classification.” Cell 178: 779–794. 10.1016/j.cell.2019.07.010 PMC6716367 31398336

[14] Balique, Fanny , Hervé Lecoq , Didier Raoult , and Philippe Colson . 2015. “Can Plant Viruses Cross the Kingdom Border and Be Pathogenic to Humans?” Viruses 7: 2074–2098. 10.3390/v7042074 PMC4411691 25903834

