# Supplementary material for: RTTAP: Empowering metatranscriptomic data analysis with a read‐based total‐infectome taxonomic solution
Source: IMetaOmics. 2025 Jul 29;2(4):e70044. doi: 10.1002/imo2.70044 (PMC12806115; doi:10.1002/imo2.70044)
Supplement: Supplementary file 1 — Supporting information. [file IMO2-2-e70044-s002.docx]

# Supporting information to RTTAP: Empowering Metatranscriptomic Data Analysis with a Read-based Total-Infectome Taxonomic Solution

**Running title**: RTTAP: A Read-based Total-infectome Taxonomic Analysis Pipeline for Metatranscriptomics

Wei Jiang^1^, Herui Liao^1^, Mang Shi^2^, Liangjun Chen^3^*, Yanni Sun^1^*

^1^Department of Electrical Engineering, City University of Hong Kong, Tat Chee Avenue, Kowloon, Hong Kong, China

^2^State key laboratory for biocontrol, Shenzhen Key Laboratory of Systems Medicine for inflammatory diseases, School of Medicine, Shenzhen campus of Sun Yat-sen University, Shenzhen, China

^3^Department of Laboratory Medicine, Zhongnan Hospital, Wuhan University, Wuhan, China

*Correspondence: chenliangjun@whu.edu.cn (Liangjun Chen), yannisun@cityu.edu.hk (Yanni Sun)

## Section A. Methods and materials

### Workflow of RTTAP

RTTAP is a short-read-based taxonomic classifier, distinguishing it from assembly-based approaches. Assembly-based methods often struggle to detect low-abundance taxa due to insufficient read overlap, preventing full read utilization and impairing contig formation. Additionally, they typically require substantial computational time and resources. In contrast, RTTAP employs a reference-based strategy that utilizes all input reads for taxonomic classification. This approach improves the sensitivity of microbial composition analysis, particularly for low-abundance organisms. As the goal is not de novo species discovery but accurate profiling of known taxa, reference-based methods provide a more efficient and practical solution for metatranscriptomic analysis.

#### Read preprocessing

In the initial stage, RTTAP preprocesses the reads by removing low-quality and host-originated reads. When raw sequencing reads are input, adapters will be removed, low-quality sequences will be trimmed and filtered, and the remaining reads will be deduplicated using fastp [15] as it is fast and integrates multiple features for NGS quality control. After quality control, host-oriented ribosomal RNA is removed by mapping quality-controlled reads to human ribosomal RNA fragments using Bowtie2 in a very-sensitive-local mode, with the reference genome set to GRCh38.p14. Unmapped reads, which indicate potential microbial reads, are retained as clean reads for subsequent taxonomic profiling.

#### Hierarchical taxonomic profiling

The second stage conducts taxonomic profiling using a combination of available methods in a hierarchical structure of two steps.

**Fast read classification into three groups** RTTAP first sorts clean reads into three microbial groups: viruses, bacteria, and fungi - quickly and accurately. RTTAP employs Kraken2 for this step, taking advantage of its strengths in terms of processing efficiency and detection sensitivity. Additionally, we derived a customized database containing only viral, bacterial, and fungal sequences in the NCBI NT database to further speed up this process while maintaining maximum sensitivity.

**Taxonomic profiling** RTTAP makes use of MetaPhlAn4 to profile bacterial reads, given its optimal trade-off between efficiency and accuracy for bacterial analysis, and uses Bowtie2 to generate alignment details of viral and fungal reads with the “-all” mapping parameter. RTTAP mitigates the multi-mapping issue by applying the LCA algorithm to merge the lineages, thereby assigning each read to the most confident taxonomic rank. For reference databases, RTTAP utilizes the NCBI viruses’ RefSeq database for virus taxonomic profiling and the EuPathDB46-Clean [16] for fungi taxonomic profiling. Additionally, RTTAP incorporates a user-configurable filtering strategy to minimize false positives from low-abundance taxa. By default, it applies a threshold of 1 reads per million (RPM) for viral taxa and 10 RPM for bacterial and fungal organisms, where RPM is calculated as the number of assigned reads in each taxon divided by the total reads in the sample, multiplied by 10^6^. These thresholds were determined based on our evaluations using both simulated and clinical datasets to strike a balance between sensitivity and specificity. An overly stringent threshold may exclude biologically meaningful signals, whereas a lenient threshold increases the risk of false positives. To accommodate diverse research needs, RTTAP provides flexibility in adjusting these thresholds. This allows users to tailor sensitivity based on analytical goals or clinical relevance. This feature enhances the specificity and interpretability of taxonomic outputs, particularly in complex metatranscriptomic datasets where background noise can obscure meaningful signals.

#### Downstream analyses

In the third stage, RTTAP provides additional features that are often of great interest to users. For bacterial reads, it employs homology and SNP models to identify ARGs using the Resistance Gene Identifier (RGI), which leverages the Comprehensive Antibiotic Resistance Database (CARD) [17]. Furthermore, RTTAP incorporates VirStrain [18] for viral strain composition analysis by utilizing the previously extracted viral reads along with a curated collection of databases containing genomes of viral strains from commonly observed pathogens.

### NGS data preparation

We prepared both simulated samples (N = 4) and real metatranscriptomic samples (N = 144) for testing and validating RTTAP’s performance. Simulated samples were generated with CAMISIM [19], containing sequences from common-seen viruses, bacteria, and fungi observed in the human respiratory tract along with human-derived sequences to simulate host background noise. Clinical respiratory samples were collected from healthy individuals, Influenza A patients, and SARS-CoV-2 patients.

### Simulated Data

To evaluate RTTAP on data with known ground-truth composition, we generated four simulated NGS datasets using CAMISIM, with NCBI RefSeq genomes as the reference. Each sample averaged approximately 13 million paired-end reads (150 bp each). Taxonomic profiles of these samples can be found in Table S4. Among these reads, 6.5 million paired-end reads originated from 50 species spanning 31 genera, including common-seen viruses, bacteria, and fungi found in the human respiratory tract. Although read counts varied across taxa in the simulated samples, their overall distribution followed a log-normal pattern. To increase the realism of the simulated samples, we spiked each dataset with ~6.5 million human-derived reads, resulting in a total of ~13 million reads per sample, closely mimicking clinical metatranscriptomic data. This human-microbial ratio was intentionally selected to evaluate the pipeline’s ability to classify microbial reads against a substantial human background while ensuring sufficient microbial signal for robust validation. After host reads removal, the effective microbial ratio increases, making this a stringent test of classification performance. Afterall, extremely low-abundant taxa (with very low RPM or read counts) are challenging for most taxonomic profilers due to their susceptibility to background noise and stochastic sampling effects.

### Clinical sample collection, RNA extraction, metatranscriptomic library preparation, and sequencing

A total of 144 respiratory samples were collected for this study, including 20 samples from healthy individuals, 62 samples from patients infected with influenza virus, and 62 samples from patients infected with SARS-CoV-2. We collected two types of specimens, pharyngeal swabs and sputum, respectively, to represent upper and lower respiratory tract infections. Specifically, among the influenza patient samples, 23 out of 62 were collected using throat swabs, while the remaining 39 samples were collected using sputum. Similarly, among the SARS-CoV-2 patient samples, 34 out of 62 were collected using throat swabs, and the remaining 28 samples were collected using sputum. All 20 samples from healthy individuals were collected using throat swabs.

After collection, all samples were stored at -80°C to preserve their integrity until further processing. Total RNA was extracted from these samples using a standardized kit provided by DAAN GENE. To account for potential contamination, four negative controls were included, consisting of throat swabs and sputum collection devices that were directly exposed to air. These negative controls underwent the same RNA extraction procedure as the clinical samples. Following RNA extraction, the concentration of the RNA samples was quantified using a Qubit 2.0 fluorometer (Invitrogen, USA). Subsequently, dual-indexed and paired-end libraries were constructed using the Trio RNA-Seq kit from NuGEN Technologies designed for low-concentration RNA samples and includes an AnyDeplete probe that removes human ribosomal RNA (NuGEN Technologies, USA). Briefly, the library preparation process involved several steps, including removal of DNA, conversion of RNA to cDNA, ligation of adapters with unique barcodes, removal of ribosomal cDNA, amplification, and library cleanup. The quality of the libraries was assessed by Qsep100 (Bioptic, Taiwan, China), and quality-controlled libraries were then sequenced on a NovaSeq platform (Illumina, USA). Four negative controls (NCs) were prepared for library construction and metatranscriptomic sequencing in parallel with the clinical samples. All sequencing files could be found in the Genome Sequence Archive (GSA) database.

## Section B. Benchmarking tools and databases for fast classification of reads into three major microbial groups

To evaluate the performance of the taxonomic profiling strategy, we employed multiple metrics shown in Equation i), ii), and iii). $N_{TP}$ denotes the number of true positive categories correctly identified. $N_{pred}$represents the total number of predicted categories at a certain level, while $N_{GT}$ indicates the ground truth categories contained in the simulated data.

$$\begin{aligned} Recall=\frac{N_{TP}}{N_{GT}}\# LISTNUM NumberDefault \end{aligned}$$

$$\begin{aligned} Precision=\frac{N_{TP}}{N_{pred}}\# LISTNUM NumberDefault \end{aligned}$$

$$\begin{aligned} F1=\frac{2\times Recall\times Precision}{Recall+Precision}\# LISTNUM NumberDefault \end{aligned}$$

Recall quantifies a tool’s ability to detect true positive categories among all ground truth categories, while precision measures the proportion of predicted categories that are actually correct. The F1-score, defined as the harmonic mean of precision and recall, provides a balanced measure of overall performance, integrating both sensitivity and specificity. This makes it particularly well-suited for evaluating the classification accuracy of our pipeline. To take genus-level as an example, the recall quantifies the percentage of correctly identified genera ($N_{TP}$) among all the ground truth genera ($N_{GT}$) present in a sample, and the precision quantifies the percentage of correctly identified genera ($N_{TP}$) among all the predicted genera ($N_{pred}$) in the results.

It is important to note that in microbial sequence classification, the number of negative instances (i.e., microbial taxa absent from a sample) vastly exceeds the number of positive instances (i.e., taxa truly present), resulting in an extremely imbalanced positive-negative ratio. Consequently, in such contexts, the False Positive Rate (FPR), whose denominator is dominated by a large pool of true negatives (TN), becomes a biased metric for pipeline evaluation and is therefore not suitable for evaluating our pipeline.

In addition, as some tools leave many reads unassigned to a taxonomic group due to limited prediction power, we used prediction rate to quantify the prediction power and evaluate the ability to identify potential microbial reads. Prediction rate is defined in Equation iv), where $n_{p}$ corresponds to the number of reads that have taxon labels assigned, and $n$ represents the total number of reads after quality control.

$$\begin{aligned} Prediction rate=\frac{n_{p}}{n}\# LISTNUM NumberDefault \end{aligned}$$

As explained in the main text, an optimal configuration of tool-database combination needs to be selected. We established a series of combinations to compare the classification performance (Table S5). These combinations included Kaiju, which utilizes its pre-built nr_eukaryotic reference database, and Kraken2, which utilizes three distinct databases: nt_microbial, nr_database, and PlusPF. Among them, PlusPF and nr_euk databases were directly downloaded from the corresponding tools’ websites; nr_database was directly indexed from NCBI BLAST NR with no modifications; nt_microbial database was created by extracting all viral, bacterial, and fungal sequences from the original NT database, considering that the NT database contains numerous sequences that were not required in our task. The size of nt_microbial database has been significantly reduced from 1.2TB (the original NCBI NT database) to 400GB, leading to a more concise database and efficient search capability.

We ran Kaiju and Kraken2 on four simulated samples using default parameters. The performance comparison can be found in Figure S1. Comprehensive reference databases (nt_microbial, nr_database, and nr_euk) demonstrated good performance, with a high category recall rate approaching 1 at both the genus and species levels (Figure S1 Category Recall). Whereas, the PlusPF database shows relatively lower average category recall (0.935 at the genus level and 0.839 at the species level) compared to the other three databases. This indicates that the PlusPF database may have limitations in sensitively identifying true-positive categories that potentially exist in the data.

Notably, all four databases demonstrated poor performance in category precision, with values below 0.05 (Figure S1 Category Precision), indicating a high rate of false-positive classifications. As a result, the corresponding category-level F1 scores were also low (Figure S1 Category F1). These results suggest that the tested taxonomic classifiers require complementary false-positive reduction strategies, as their outputs contain an excessive number of false positive results, severely compromising classification reliability.

As for the prediction ability, prediction rate shows the ability to identify potential microbial reads in a sample. The combination of Kraken2 + nt_microbial database presents the highest prediction rate among the four combinations (Figure S2). As we move from the microbial level to the genus and species levels, there is a noticeable decrease in the prediction rates. However, the combination of Kraken2 + nt_microbial database consistently demonstrates the highest prediction rate among the four combinations. On average, it achieves a prediction rate of 0.92, 0.83, 0.6 at the microbial, genus, and species level, respectively. This suggests that the nt_microbial database is particularly effective in capturing and classifying a broad range of microbial taxonomies.

In terms of run times, Kraken2 ran much faster compared to Kaiju, as shown in Figure S3. It achieved an average run time of approximately 2 minutes per sample when applied to the four simulated datasets we generated. In contrast, Kaiju required an average run time of 21 minutes per sample to classify the same simulated databases. The relatively slow speed of Kaiju may be attributed to the translation process of nucleotide sequences into protein sequences. Furthermore, Kraken2 distinguishes itself in terms of speed due to its kmer-based method, which significantly accelerates the indexing and mapping processes, making it a swift and efficient choice for the initial stage of taxonomic profiling.

In the initial stage of taxonomic profiling, our priority is high recall performance, which maximizes the identification of true positive categories and allows for the prediction of a greater number of reads. Additionally, we value fast processing speed as a desirable characteristic. Based on these criteria, we have determined that Kraken2 paired with the nt_microbial database is the optimal choice for the first stage of RTTAP, which can efficiently and accurately identify microbial reads.

## Section C. Interpretation of clinical results

### Taxonomic profiling

Among the 20 samples from healthy individuals, RTTAP detected few pathogens, indicating a low false-positive rate. To assess its sensitivity, we next examined its performance in clinically confirmed cases. RTTAP exhibited strong diagnostic performance, detecting Influenza A virus in 95% (59/62) of clinically confirmed influenza cases. The pipeline showed slightly reduced but still substantial sensitivity for SARS-CoV-2, with 68% (43/62) detection in COVID-19 patient samples. While most positive samples showed high viral read counts (RPM), three potential factors accounted for undetected cases: (1) stringent abundance thresholds excluding extremely low-abundance viruses (< 1 RPM), (2) late-stage SARS-CoV-2 infections with viral loads below detection limits, and (3) pre-analytical variability in sample processing. Furthermore, it’s important to note that metatranscriptomic datasets only capture species actively replicating at the time of sampling, potentially overlooking latent or non-replicating pathogens. These detection patterns reflect both the analytical sensitivity of our pipeline and the biological reality of variable viral loads in clinical specimens. Notably, several samples revealed the presence of multiple virus species. For instance, sample “SZ019QC” contained both *Influenza A virus* and *Human respiratory syncytial virus A*. This finding suggests the possibility of co-infection by different viruses within these samples, which may require careful attention when managing such clinical cases.

Given the limited number of species detected across all samples, and that most patient samples contained only one single pathogenic fungal species, the Shannon diversity index was considered unsuitable for characterizing the fungal communities across samples. Instead, we presented the mean relative abundance (RPM) of each pathogenic fungal species within each sample group (main text Figure 1G). Additionally, we calculated alpha diversity (species richness) for the pathogenic fungi and found that samples collected from SARS-CoV-2 patients harbored a greater variety of pathogenetic fungi species compared to the other two groups (main text Figure 1G, Kruskal-Wallis test, *p* < 0.0001; Dunn's post-hoc test, Healthy: *p* = 1.74 × 10^-6^; Influenza A: *p* = 1.233 × 10⁻^9^). Furthermore, we conducted statistical tests to evaluate differences in fungal species abundance across sample groups and denoted statistically significant differences with asterisks. Notably, *Candida albicans* exhibited a significantly higher relative abundance in SARS-CoV-2 samples (Kruskal-Wallis test, *p* < 0.0001; Dunn's post-hoc test, Healthy: *p* = 3.643 × 10⁻^5^; Influenza A: *p* = 6.885 × 10⁻^8^). This makes *C. albicans* the dominant fungal species in SARS-CoV-2 patients, suggesting a potential association with secondary fungal infections in these individuals. However, this hypothesis requires further experimental validation.

### ARG profiling

The ARGs were grouped into various drug categories based on the antibiotic resistance information provided by RGI in conjunction with the CARD database. Among all the ARGs found in these samples, over half of them exhibit resistance to multiple classes of drugs (Figure S7). Notably, *cephamycin resistance genes presents the highest prevalence among single-drug resistance genes*, indicating a widespread resistance of *cephamycin* among all types of samples. ARGs highlighted in red font indicate that their observed pathogens (according to RGI’s results) overlap with taxa present in taxonomic profiles. Star symbols on the heatmap denote instances where ARGs co-occurred with their known bacterial carriers in individual samples. These ARG-bacterium co-occurrences warrant special attention, as they may potentially transfer antibiotic resistance to clinically relevant pathogens identified in the taxonomic profiles.

### Viruses strain-level profiling

While viral sequences were detected at the genus level in some healthy controls, no pathogenic virus strains were identified at the species or strain level in these 20 samples. (Figure S8). In contrast, out of the remaining 124 samples collected from influenza and SARS-CoV-2 patients, a majority displayed positive results for pathogenic virus strains, particularly Influenza A virus strains and SARS-CoV-2 virus strains.

Influenza A virus strains displayed a wide range of abundance, with strain depths ranging from 3.409 to 9358.159 across 62 samples obtained from influenza patients. Strain depth refers to the sequencing coverage and reads support for identified viral strains, calculated as the average depth of strain-specific variant positions, and can reflect the relative abundance of each strain. The most prevalent and abundant influenza virus strain in our samples is *Influenza A virus A/Hawaii/07/2019*, while accompanied by other Influenza A strains such as *Influenza A virus A/Hawaii/31/2018*, *Influenza A virus A/California/91/2018*, and *Influenza A virus A/Utah/15/2019*. These findings indicate a widespread circulation of Influenza A viral strains around 2019. Interestingly, some of the identified strains were from 2020, which precedes the sampling period. This anomaly could be attributed to a delay in the identification, characterization, and naming processes for these particular strains.

Similar patterns are observed with SARS-CoV-2. The abundance of SARS-CoV-2 strains demonstrated a broad range, with strain depths varying from 3.607 to 98206.927. Dominant strains, such as *hCoV-19/Kazakhstan/KZ_Almaty/2020* and *hCoV-19/Yunnan/IVDC-YN-003/2020*, are readily identifiable from the figure. Such information is of great help for tracing the origins of the disease and for understanding the epidemiology of the virus.

## References

1. Chen, Shifu, Yanqing Zhou, Yaru Chen, Jia Gu. 2018. “fastp: an ultra-fast all-in-one FASTQ preprocessor.” *Bioinformatics* 34: i884−i890. <https://doi.org/10.1093/bioinformatics/bty560>

2. Lu, Jennifer, Steven L. Salzberg. 2018. “Removing contaminants from databases of draft genomes.” *PLOS Computational Biology* 14: e1006277. <https://doi.org/10.1371/journal.pcbi.1006277>

3. Alcock, Brian P., William Huynh, Romeo Chalil, Keaton W. Smith, Amogelang R Raphenya, Mateusz A. Wlodarski, Arman Edalatmand, et al. 2023. “CARD 2023: expanded curation, support for machine learning, and resistome prediction at the Comprehensive Antibiotic Resistance Database.” *Nucleic Acids Research* 51: D690−D699. <https://doi.org/10.1093/nar/gkac920>

4. Liao, Herui, Dehan Cai, Yanni Sun. 2022. “VirStrain: a strain identification tool for RNA viruses.” *Genome Biology* 23: 38. <https://doi.org/10.1186/s13059-022-02609-x>

5. Fritz, Adrian, Peter Hofmann, Stephan Majda, Eik Dahms, Johannes Dröge, Jessika Fiedler, Till R. Lesker, et al. 2019. “CAMISIM: simulating metagenomes and microbial communities.” *Microbiome* 7: 17. <https://doi.org/10.1186/s40168-019-0633-6>

## Supplementary figures


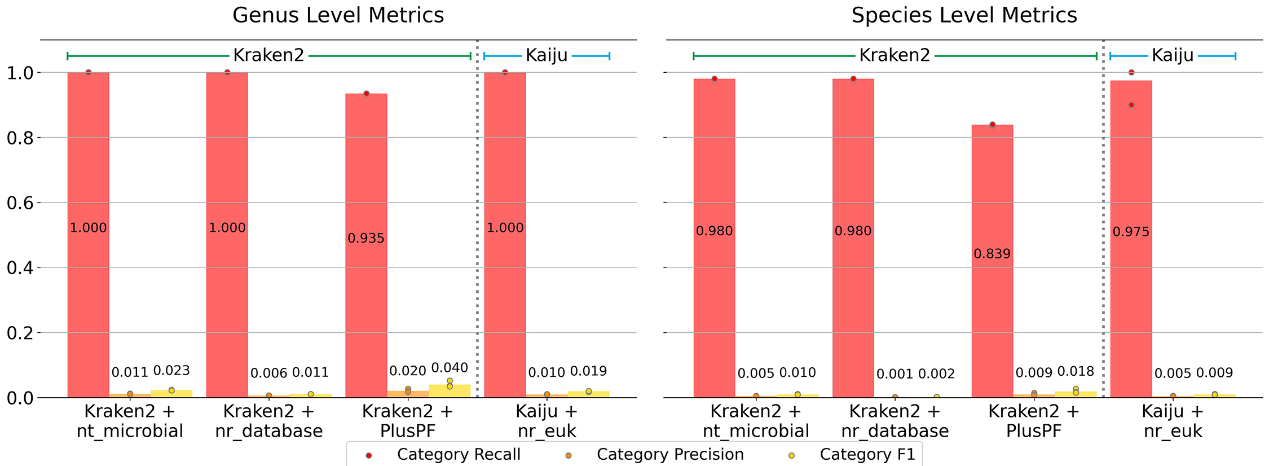


**Figure S1.** **Category recall, precision, and F1 of four combinations at the genus and the species level.** X-axis: tool and reference database combination; Y-axis: category recall/precision/F1 value. Dots within a bar represent the performance of a combination on four simulated samples (one dot per sample). The height of each bar and the text in them present the mean value across the four data points.


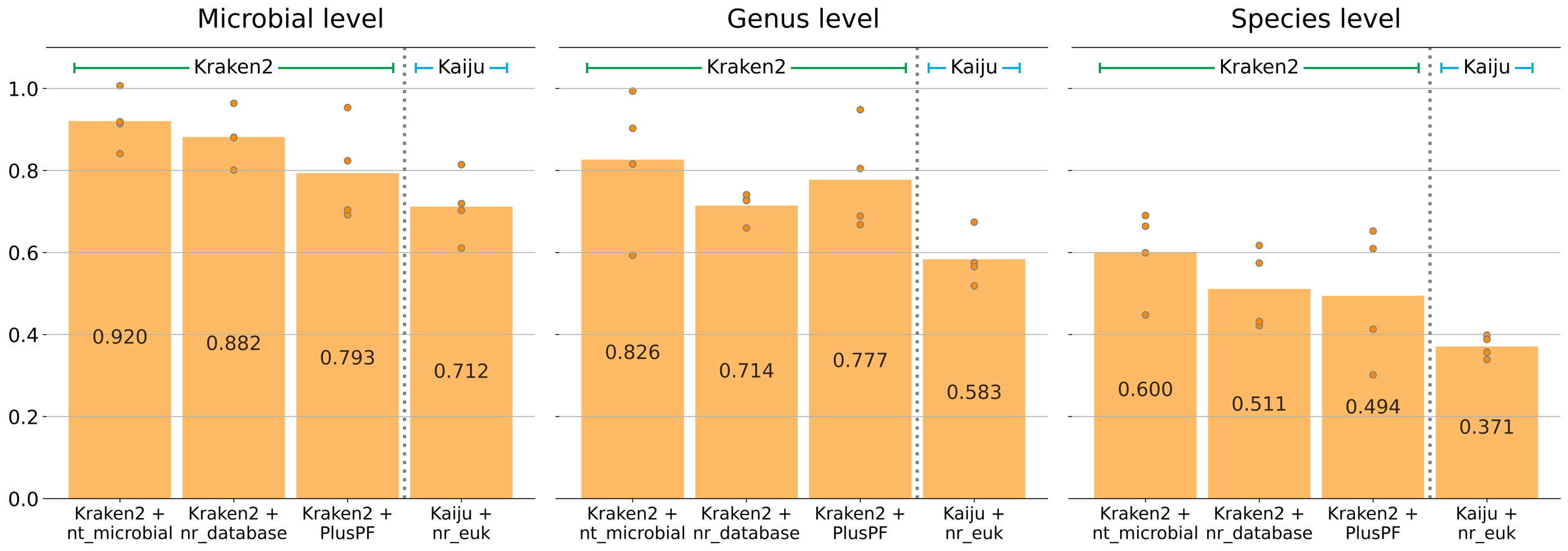


**Figure S2. Prediction rate of four combinations across different rank levels.** X-axis: combinations of tools and reference databases; Y-axis: prediction rate. Dots within a bar represent the performance of a combination on four simulated samples (one dot per sample). The height of each bar and the text in them present the mean value across the four data points.


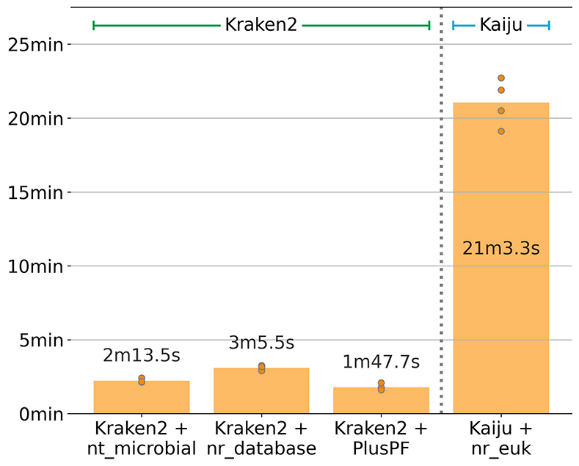


**Figure S3.** **Run time of different tool-database combinations.** X-axis: tool and reference database combination. Y-axis: run time in minutes. Each dot represents the performance of a combination on a simulated sample. The height of each bar and the text within the bar indicate the mean value of the combination’s run time across the four simulated samples.


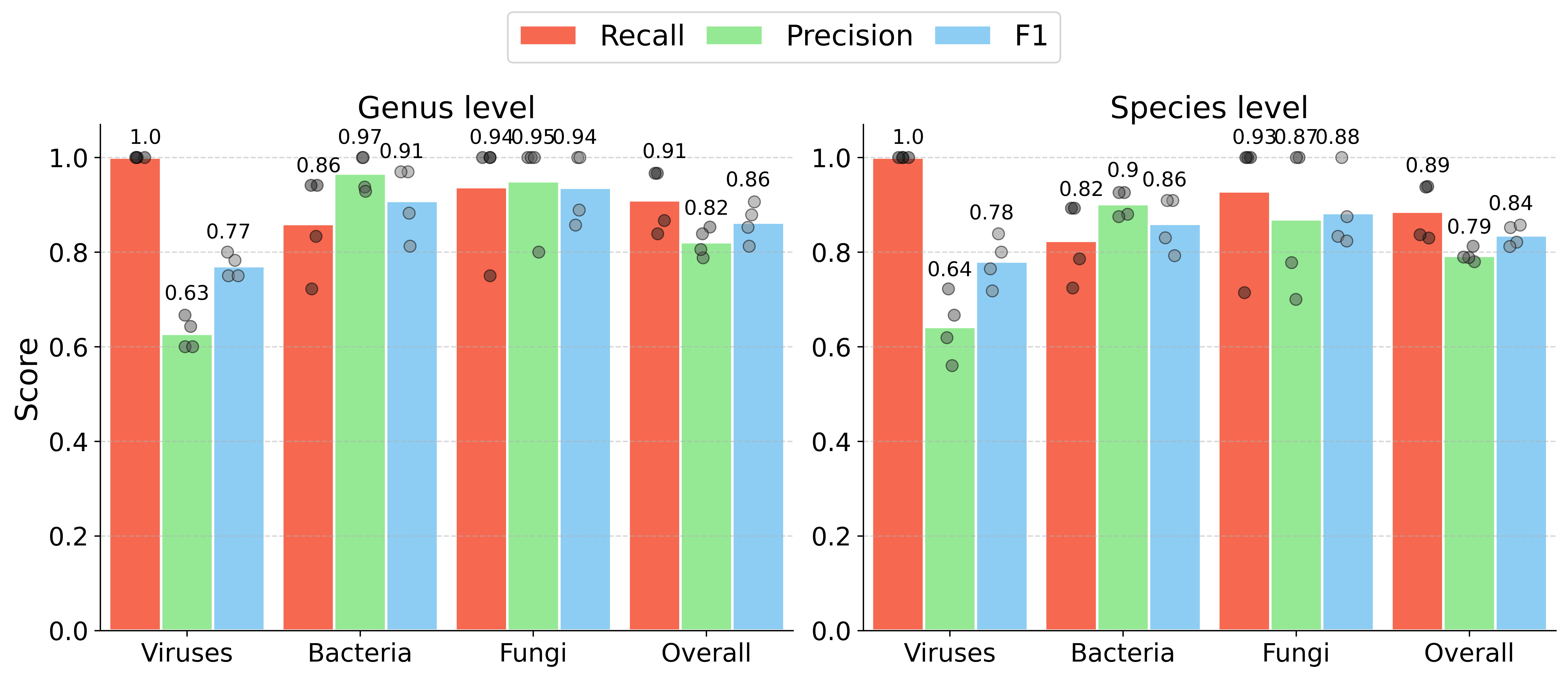


**Figure S4. RTTAP’s performance in identifying viral, bacterial, and fungal reads.** X-axis: groups. Y-axis: category recall/precision/F1 value. The bars and texts in them depict the mean values of the corresponding metrics. The scatter dots represent the metrics of each sample.





**Figure S5. Full view of genus level taxonomic profiles.** Column: a clinical sample. Row: genus followed by taxid. The abundance is presented in logarithmic RPM with base 10. Bars on the left represent different groups of genus-level categories. Bars on the top represent different sources of samples.





**Figure S6. Full view of species level taxonomic profiles.** Column: a clinical sample. Row: species followed by taxid. The abundance is presented in logarithmic RPM with base 10. Bars on the left represent different groups of species-level categories. Bars on the top represent different sources of samples.





**Figure S7. ARG abundance and co-occurrence of 144 clinical human samples.** Column: a sample. Row: ARG name. The green bar on the left displays ARG abundance on a base-10 logarithmic scale. The bars on the right provide categorical information about the ARGs: the left bar indicates the bacterial species both co-occur with and known to carry each ARG, while the right bar indicates the corresponding resistant drug classes.


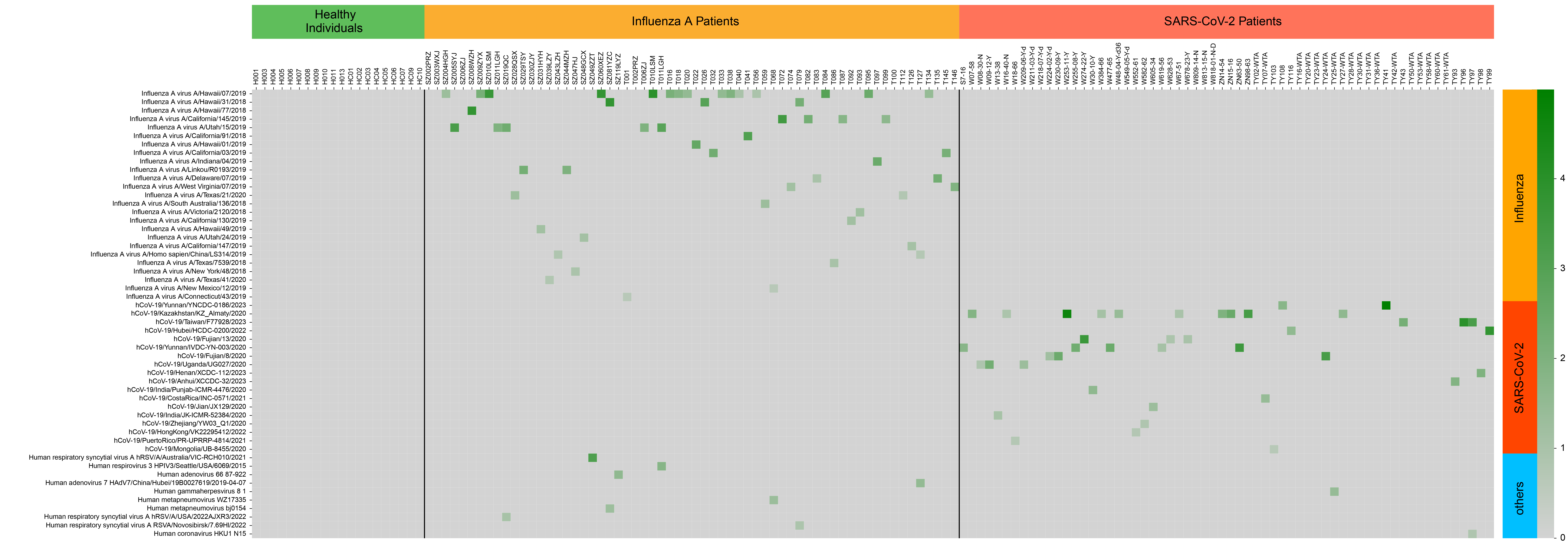


**Figure S8. Viral strain-level profile of 144 clinical human samples.** Row: virus strain. The color intensity indicates the depth of each strain in logarithmic scale with base 10. Bars on the left indicate the associated species of these viral strains. Bars on the top represent different sources of samples.
